# Quantity and distribution of arbuscular mycorrhizal fungal storage organs within dead roots

**DOI:** 10.1007/s00572-016-0741-0

**Published:** 2016-11-12

**Authors:** Anja Müller, Benard Ngwene, Edgar Peiter, Eckhard George

**Affiliations:** 1 Leibniz-Institute of Vegetable and Ornamental Crops, Theodor-Echtermeyer-Weg 1, 14979 Grossbeeren, Germany; 2 Plant Nutrition Laboratory, Institute of Agricultural and Nutritional Sciences (IAEW), Faculty of Natural Sciences III, Martin Luther University Halle-Wittenberg, 06099 Halle (Saale), Germany; 3 Department of Crop and Animal Sciences, Plant Nutrition, Humboldt University, Unter den Linden 6, 10099 Berlin, Germany

**Keywords:** Spore production, Vesicles, Extraradical mycelium, Root colonisation, Root turnover

## Abstract

The formation of storage organs, such as spores and vesicles, is a central part of the life cycle of an arbuscular mycorrhizal fungus (AMF), but the conditions under which this occurs in AMF are not well understood. Here, quantity and distribution of storage organs formed by the arbuscular mycorrhizal fungus (AMF) *Funneliformis mosseae* within dead (excised) roots were characterised. ‘Trap roots’ (TR), separated from the growth substrate by a 30-μm mesh, supported hyphal growth and formation of storage organs of the AMF. Hyphae developed both inside and on the outside of the TR and also within air gaps of surrounding nylon mesh compartments, but formation of vesicles and spores was confined to the interior and to the surface of the TR. Up to 20 % of the TR length harboured newly formed storage organs, resulting in a number of about 60 per mg TR dry weight. The portion of TR length containing storage organs was greater in coarse (diameter >300 μm) than in thin (<150 μm) TR, irrespective of whether the TR were sourced from an AMF host or non-host plant. We conclude that the AMF’s extraradical mycelium produces its storage organs within dead roots in preference to air space in the substrate. Dead roots may indirectly supply nutrients to AMF (once they have been mineralised) or represent a protected space for the fungal structures to develop. The experimental technique described here allows for the preparation of AMF spores and vesicles of *F. mosseae* free of any mineral substrate.

## Introduction

Arbuscular mycorrhizal fungi (AMF) form a symbiotic association by colonising the cortical cells of host plant roots. The symbiosis is not restricted to the space within the root (the ‘intraradical space’), since the AMF also develops extraradical mycelium (ERM), which allows it to spread within the soil. The important storage organs for AMF are spores which contain storage lipids and are protected by a multi-layered cell wall allowing them to remain viable for several years and thus being crucial for the establishment of new colonies (Brundrett 1991). Also, vesicles are thick-walled lipid-storing organs and can, for some AMF species, serve as propagules. In the following sections, spores and vesicles are summarised under the term ‘storage organ’ in cases where a differentiation between both was not possible.

To a large extent, AM fungal development is governed by the nutritional status of the host plant. A low phosphorus supply to the plant (while other nutrients are not limited) can promote fungal root colonisation, intensify ERM development and also encourage spore formation (Douds 1994). During symbiosis, AMF spore production is regulated by the supply of carbon derived from its host (Ijdo et al. 2010). However, with respect to the colonisation strategy adopted by different AMF species, there is a variation (Varela-Cervero et al. 2016). For example, members of the suborder *Glomineae* can initiate the symbiosis from spores, vesicles or hyphal fragments, while most members of *Gigasporineae* rely exclusively on spores (Klironomos and Hart 2002).

In addition to sporulating within the mineral part of soils, some AMF species have also been observed to sporulate within synthetic air-filled voids such as glass capillaries (Rydlova et al. 2004), in empty seed cavities (Taber 1982), in dead AMF spores (Koske 1984), in nematode cysts (Francl and Dropkin 1985) or in nodules of legumes (Vidal-Dominguez et al. 1994). AMF vesicles have been observed within the tissue of decomposing plant leaves (Aristizábal et al. 2004). A high number of AMF storage organs was found inside root fragments present in commercial inocula containing propagules of *Rhizophagus irregularis* and *Funneliformis mosseae* (own unpublished observation). It is not clear, however, whether these organs have developed in living root tissue or whether they were formed after death of the host plant. The factors which stimulate or induce spore and vesicle formation inside air-filled voids are not known. Rydlova et al. (2004) suggested that the ability of AMF to sporulate inside empty seed cavities has evolved as a strategy to escape predation from soil arthropods. Dead roots are ubiquitous in soil, both in natural habitats and in agricultural fields as post-harvest residues, which may also form nutrient-rich patches in the soil. Organic residues with a low carbon-to-nitrogen ratio, such as the dead roots of annual plants, represent a readily mineralisable source of nutrients for soil bacteria (Högberg et al. 2007), many of which are associated with AMF (Bianciotto et al. 1996).

AMF inocula are commonly produced in substrate-based systems and less often in hydroponics, aeroponics and in vitro cultures (Ijdo et al. 2011). In substrate-based inocula, AM fungal propagules and hyphae (located within and on the surface of host roots and in the surrounding growth substrate) are mixed together with a growth substrate, such as quartz sand. The main disadvantage here is that it is difficult to remove the attached substrate (containing nutrients and other organisms than AMF) when studying the AM fungal mycelium. A strategy to overcome that could be trapping AMF fungal structures in substrate-free media such as plant root fragments (‘trap roots’, TR) that are available, for example, from hydroponic plant production.

In order to select for the most suitable TR species for the accumulation of AMF structures, it is necessary to investigate AM fungal spread in roots of different genotypic origin and geometry. A detailed quantification of formed AMF storage organs inside dead root fragments was not yet available in literature, and this question is addressed here in the form of two specific objectives. The first was to assess how the source of the root fragments would affect the quantity of intraradical storage organs formed. Therefore, dead roots were obtained from nasturtium (*Tropaeolum majus* L.) and pak choi (*Brassica rapa* L. ssp. *chinensis*), chosen as representatives of host and non-host plant species that produce a range of anti-fungal glucosinolates (Verkerk et al. 2009), to test if AMF abundance would be reduced in such dead roots. Additionally, dead roots of tomato wildtype and mycorrhiza-defective tomato served as representatives of vegetable plants (host and non-host) without any anti-fungal compounds. The second objective was to test whether the root diameter has an effect on the quantity of storage organs formed. This notion derived from the suggestion that the air space formed within a decomposing root attract AMF proliferation (Rydlova et al. 2004). The hypothesis here was that a coarser root material would lead to an increase in the formation of AMF storage organs.

## Materials and methods

### Maize plant pre-cultivation and AM fungal inoculation

**Fig. 1:**
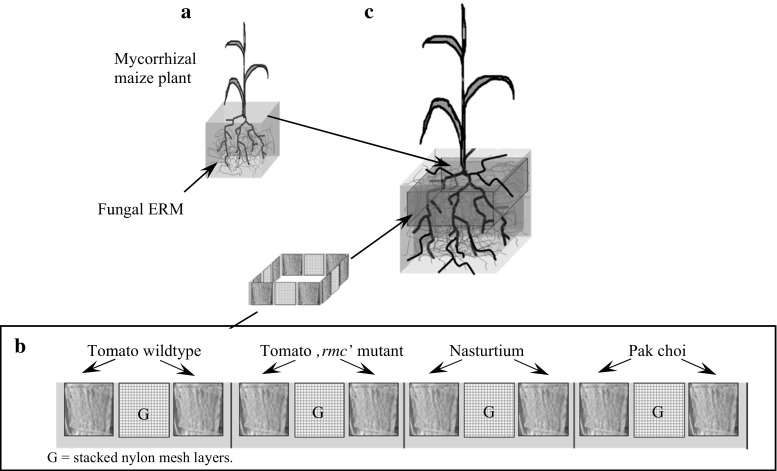
Illustration of the experimental set-up for the TR bags used. **a** Pre-cultivated, AMF-inoculated maize plant. **b** Compartmentalised TR bag containing TR originating from different plant sources (as indicated) or control filling (stacked nylon mesh layers; *G*). **c** AMF-colonised maize plant after transfer to a larger pot. The upper part of its root system is surrounded by a TR bag

AM fungal inoculated maize plant root systems were established for later insertion of tested trap roots. Seeds of *Zea mays* (L.) ‘Golda’ were germinated in the dark on cellulose filter paper soaked in saturated CaSO_4_ solution. At the stage when the first leaf was fully expanded, single seedlings were planted into 1-L plastic pots (TEKU-Tainer; Pöppelmann, Germany) containing 1.3 kg dry substrate (Fig. 1a). The growth substrate—a loamy sand with 45.2 % sand, 42.0 % silt and 12.8 % clay—was obtained from the C-horizon of a Luvisol at Weihenstephan, Germany (48° 25′ N, 11° 50′ E). To eliminate any pre-existing AMF propagules, the substrate was dry-heated twice for 24 h at 85 °C. The substrate contained 5.2 and 3.4 mg kg^−1^ CaCl_2_-(0.0125 M)-extractable NH_4_^+^ and NO_3_^−^, respectively. It also contained 6.5 mg kg^−1^ of acetate-lactate-extractable (CAL; see Schüller (1969) P and 65.7 mg kg^−1^ of CAL-extractable K. The CAT-extractable micronutrients (CaCl_2_/DTPA; see Alt and Peters 1993) were measured as 15.0 mg kg^−1^ Mn, 0.3 Zn mg kg^−1^ and 0.9 mg kg^−1^ Cu. The organic matter content was measured as 0.3 % (*w*/*w*), and the pH (CaCl_2_) was 7.7. The substrate was fertilised with 200 mg K (K_2_SO_4_), 100 mg N (NH_4_NO_3_), 100 mg Mg (MgSO_4_), 50 mg P (KH_2_PO_4_), 10 mg Fe (Fe-EDTA), 10 mg Cu (CuSO_4_) and 10 mg Zn (ZnSO_4_) per kg of dry substrate. The nutrients were solubilised in deionised water before mixing into the substrate. AM fungal inoculation was done by mixing the substrate with 10 % (*w*/*w*) of a quartz-sand-based inoculum containing propagules of *Funneliformis mosseae* (formerly *Glomus mosseae*) supplied by INOQ GmbH (Schnega, Germany). Fourteen replicated pots were produced this way. After planting, the substrate moisture content was maintained at 18 % (*w*/*w*) using deionised water. Water loss was measured weekly by weighing. The plants were grown under greenhouse conditions for 49 days in April and May. Over the growing period, the day/night temperatures averaged 24/19 °C, and the mean relative air humidity was 64 %.

### Production and preparation of trap root material

To obtain TR material, tomato (*Solanum lycopersicum* L.) wildtype cv. RioGrande 76R, its derived mycorrhiza-defective ‘*rmc*’ mutant (Barker et al. 1998), nasturtium (*Tropaeolum majus* L.) and pak choi (*Brassica rapa* L. ssp. *chinensis*) were all germinated in the dark on cellulose filter paper soaked in saturated CaSO_4_ solution. The seedlings were transferred to an aerated hydroponic solution (pH 6.8) containing 5 mM N, 0.7 mM P, 4 mM K, 2.5 mM Ca; 1 mM Mg, 4 mM S, 10 μM Fe (as Fe-EDTA), 10 μM B, 5 μM Mn, 1 μM Zn, 0.7 μM Cu and 0.05 μM Mo, with the solution being renewed twice a week. Following the growing period of 7 weeks, roots were harvested and representative samples of 1 g (fresh weight) were taken and cut into approximately 2-cm-long fragments. The mean root length of the sample (four replicates per plant species) was determined by light microscopy using a modified line intercept method (Newman 1966) with 50-fold magnification with a dissecting microscope. The mean root diameter was measured from a sample of 20 root fragments per replicate using the hairline micrometer located in the ocular of a compound microscope (400-fold magnification). To calculate the specific trap root length per unit dry weight (Table 1), the samples were dry-heated at 60 °C for 48 h and subsequently re-weighed.

**Table 1:** Properties of the TR material prior to experimental use. Values were given as means ± standard deviation

TR origin	Average TR diameter (μm)	Specific TR length (m g^−1^ DW)
Tomato wildtype	332 ± 120	65 ± 11
Tomato ‘*rmc*’ mutant	313 ± 137	66 ± 8
Nasturtium	338 ± 122	53 ± 6
Pak choi	171 ± 68	130 ± 21

To ensure that no pre-existing AMF or other fungal contaminations were present, the roots were analysed microscopically prior to dry-heating. Four 1-g samples of the freshly harvested root material were stained with trypan blue following the method of Koske and Gemma (1989), and the extent of AMF colonisation was determined using a modified gridline intersection method (Tennant 1975; Kormanik and McGraw 1982) with 50-fold magnification, counting 150–300 intersections per sample. All root material from these solution cultures was confirmed to be free from any fungal colonisation.

### Construction and filling of TR compartments

Prior to their experimental use as trap roots, freshly harvested root material was cut into approximately 2-cm-long fragments, sterilised by immersion in 70 % (*v*/*v*) ethanol for 30 s, blotted dry with paper towels and then left to evaporate for 5 min.

The TR bags were made from two layers of a 30-μm-mesh nylon membrane, which allowed access for AMF hyphae but prevented plant roots from passing through. Each compartment bag was 52 cm (length) by 4 cm (width). About 1-cm-wide strips of silicone (Probau, Bauhaus AG, Germany) were used to seal the edges of the bags and to separate them into 12 similarly sized (3 × 4 cm) compartments (Fig. 1b). The 52-cm-long compartmented TR bag fitted around the circumference of the transplanted maize plant root system. Each compartment was filled with fresh TR material with an amount equal to 100 mg dry weight, originating either from the tomato wildtype (WT), tomato mutant (*rmc*), nasturtium or pak choi, for which an amount equal to 50 mg dry weight was used, due to its higher specific root length (see Table 1). To test whether storage organ formation and hyphal growth within the compartment may be restricted to or preferably occur in the air space within the TR compartment, nylon mesh traps were created by stacking three layers of a nylon mesh (2-mm-mesh size; 3 × 4 cm) and sealing it in the same way as was done for the TR-filled compartments. After the filling process, the final thickness of each compartment was approximately 3 mm, irrespective of whether the filling was TR material or nylon meshes.

### Experimental set-up

The 49-day pre-cultivation period of the AMF-inoculated maize plants served to establish AMF root colonisation. In order to create the treatment with inactivated AM fungi for the following experiment, four of the prepared replicated pots were randomly chosen to remove the maize plants shoot 1 day prior to the TR insertion (‘maize shoot removal’ treatment). The remaining replicated pots were prepared as mycorrhizal treatment (AM_v_), where the plant shoot remained intact. To introduce the TR bags, each maize plant root system was removed from the former planting pot and the upper 4 cm were surrounded with one TR bag each. The wrapped root system was then placed in the centre of a larger 2 L pot containing 2-kg heat-sterilised (85 °C, 48 h) substrate, prepared and fertilised as described above. The gaps between root system and pot wall were filled with part of the 2-kg substrate (Fig. 1c). The filled TR bags were added to the experimental pots immediately (less than 1 h) after obtaining the TR from the plants.

The pots were irrigated twice weekly to maintain a substrate moisture content of 18 % (*w*/*w*). The TR compartments remained in the pots for another 14 days of maize plant cultivation, before the experiment was harvested.

### Harvest and quantification of AMF structures

After removal of the TR compartments from the pots, TR material was carefully extracted from the bags. The maize roots were washed free of substrate. Mean TR length per compartment (four replicates per plant species), average TR diameter and specific length of TR were determined after harvest, as described above.

Maize plant roots and TR material were stained with trypan blue; then the percentage of root length with AM fungal intraradical storage organs (i.e. vesicles and/or spores) and hyphae (Fig. 3a) was quantified using a modified gridline intersection method, as described above. The stacked nylon meshes were also scanned for AMF structures; to minimise any loss of AM fungal structures, the nylon meshes were left inside the bags during the staining process and were stored in lactic acid after staining.

The number of AMF intraradical storage organs per unit TR length (Fig. 4a) was determined by mounting fragments of TRs on microscope slides; for each TR replicate sample, the number of intraradical storage organs within 20 randomly chosen 3-mm segments of the TR was counted. The length of the observed TR segment was measured using a hairline micrometer located in the ocular of the compound microscope; at the same time, the diameter of the AMF storage organ was recorded. The number of intraradical storage organs per centimetre TR length (S_TRcm_) was calculated as S_TRcm_ = 10/3 × *n* × S_TR_, where *n* is the average number of intraradical storage organs per 3-mm segment and S_TR_ is the percentage of TR length with storage organs. Additionally, the S_TRcm_ was translated to number of storage organs per TR volume in cm^3^ using the average TR diameter (Fig. 4b). Using the specific root length of TR after harvest (spec_rl_ in cm mg^−1^ dry weight), number of storage organs per milligramme TR dry weight (Fig. 4a) was calculated as S_TRmg_ = S_TRcm_ × spec_rl_.

The percentage of TR length, either non-colonised or colonised with AMF intraradical storage organs, was quantified separately for the three root diameter classes <150 μm (thin), 150–300 μm (intermediate) and >300 μm (coarse). Four replicates of each species of TR were evaluated, comprising three pooled TR compartments each (combined from different maize plant pots). The samples were evenly spread on a microscopic slide with an underlying 5-mm grid, and the crossing point of a root over a grid line was taken as the measurement point. At the same position, the individual root diameter was measured using a hairline micrometer located in the ocular. For each sample, about 150 intersections (at least 20 per root diameter class) were observed.

The spore number per unit substrate volume was quantified after four 200-g samples of the substrate were taken and the AMF spores were extracted using the wet-sieving and decanting method (Gerdemann and Nicolson 1963), whereby the material was laid over a 70 % sucrose solution and centrifuged (2000 rpm, 2 min). After staining in trypan blue for 24 h, the spores were attached to a gridded nitrocellulose membrane (Hanssen et al. 1974) for counting.

### Statistical analyses

Raw data with a normal distribution (Kolmogorov–Smirnov test; *P* > 0.05) and homogeneity of variance (Levene’s test; *P* > 0.05) were subjected to a one-way analysis of variance. The multiple comparison Tukey’s test was used to assign significant differences between means, applying a cut-off of *P* < 0.05. Data which were not normally distributed were subjected to the Kruskal–Wallis test (*P* < 0.05). All statistical calculations were conducted using routines implemented in SPSS v 15.0 software (SPSS Inc., Chicago, IL, USA).

## Results

### Presence of AMF structures within the TR

**Fig. 2:**
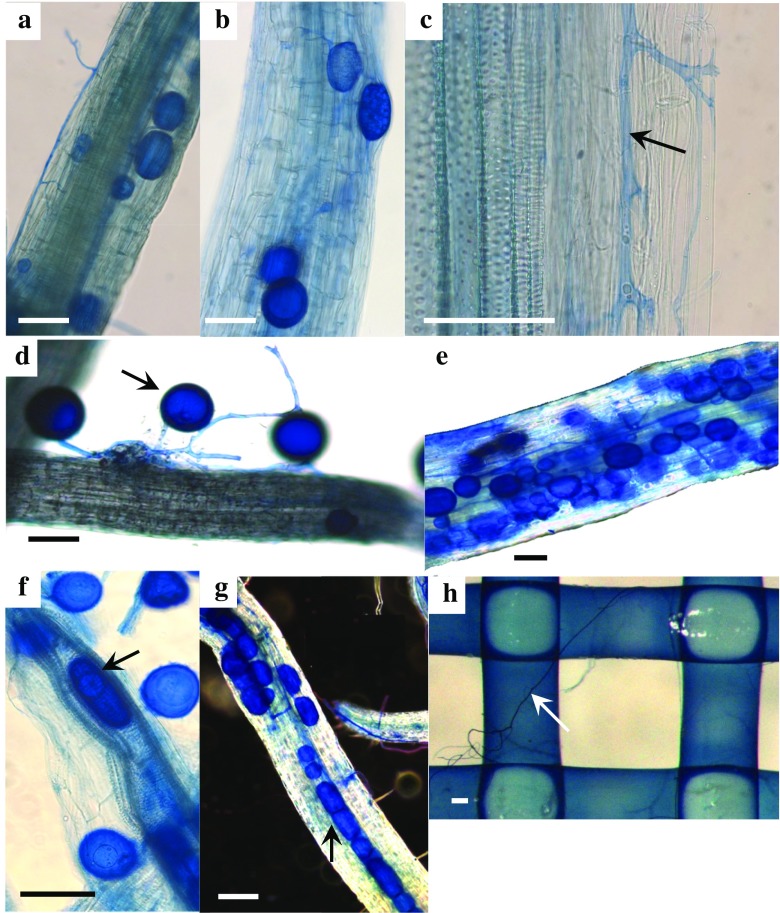
Microphotographs of trap roots (TR) at the end of the experiment. TR have been placed in pots for a 14-day period, separated by a membrane from neighbouring AMF-inoculated maize roots. Hyphae and storage organs within the cortex of **a** the ‘*rmc*’ tomato mutant and **b** nasturtium; **c** intercellular growth of hyphae along the cortex of nasturtium; **d** pak choi TR of diameter <150 μm colonised by AMF on the outer surface; **e** coarse TR containing AMF storage organs; **f**, **g** vascular cylinders harbouring AM fungal ovoid and globose-shaped storage organs, shown in a dissected (**f**) and in an intact TR (**g**); **h** top view of a nylon mesh layer, sampled from a stacked nylon mesh control. *Bars* 100 μm

This study aimed to evaluate the growth of AMF extraradical mycelium and formation of AMF storage organs (including vesicles and spores) within and on the surface of trap roots (TR) originating from either host (tomato and nasturtium) or non-host plant species (a mycorrhiza-defective tomato mutant and pak choi). In all cases, maize plant roots were successfully prevented from entering the TR compartments: none of the roots have crossed any of the TR compartment membranes. In treatments with viable AMF (AM_v_), it was found for all the TR origins that AM fungal hyphae had diameters of 3 to 15 μm and were present both on the TR surface and along its main axis (Fig. 2a), as well as internally between cortical cells (Fig. 2c). The AMF developed thick-walled, oval or globose storage organs (diameter of about 50 to 100 μm) within the TR cortex of different TR origins (Fig. 2a, b, e). Some globose storage organs (here spores) were also deposited on the outer surface of the TR, in most cases observed with TR of pak choi (Fig. 2d). Ovoid and globose-shaped storage organs were also formed within the vascular cylinders of coarse roots (Fig. 2f, g). In compartments filled with nylon meshes, hyphae were observed at the nylon mesh surfaces and also passed through the mesh layers, but hyphae did not form storage organs at these points (Fig. 2h). No AMF arbuscules were present inside the TR. In the ‘maize shoot removal’ treatments, TR contained no fungal structures.

### Quantities of AMF structures in the TR

**Fig. 3:**
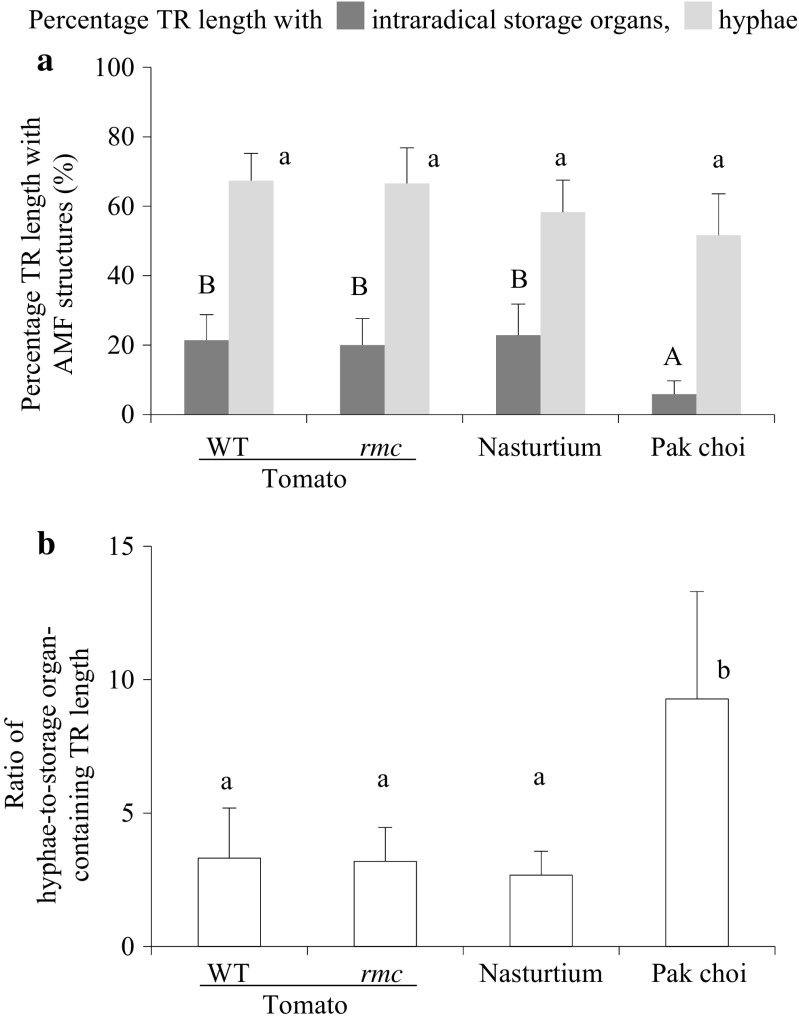
Percentage of TR length with AMF structures and hyphal-to-storage organ ratio. Percentage of TR length with **a** AMF hyphae (grown longitudinal to the TR main axis, located in- and outside the root cortex) and intraradical AMF storage organs. **b** Ratio of hyphae-to-storage organ-containing TR length. Values are given as means ± standard deviation. *Bars topped by the same letter* do not differ significantly (*P* < 0.05; *n* = 4). *Capital letters* belong to the statistical analysis of data on intraradical storage organs

**Fig. 4:**
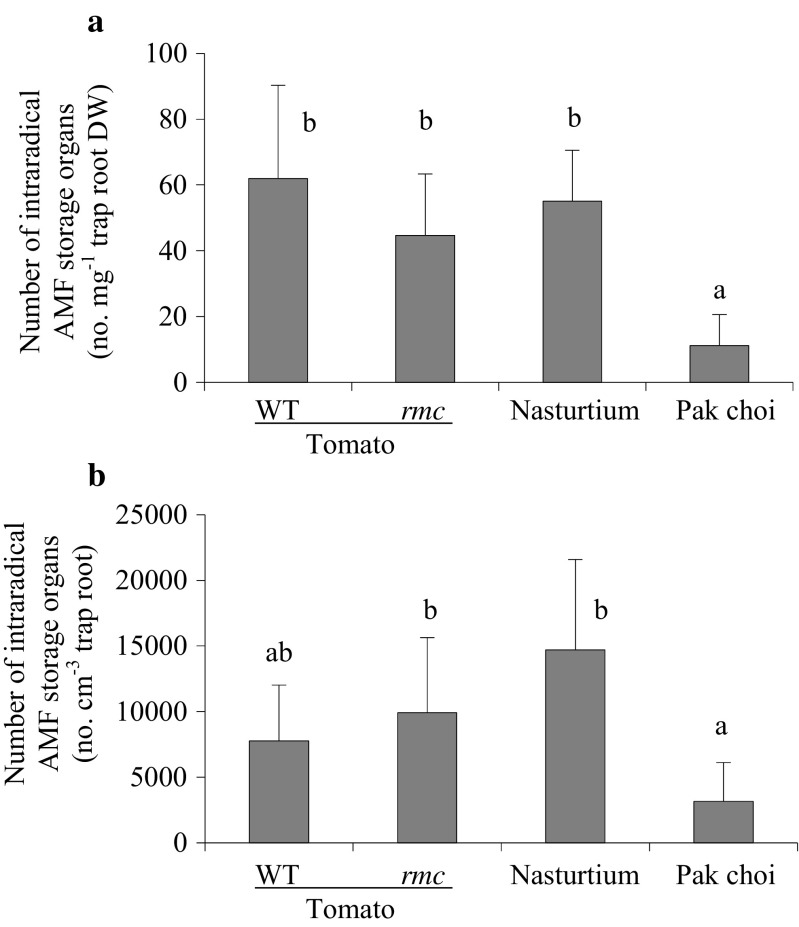
The number of intraradical AM fungal storage organs **a** per milligramme TR dry weight (DW) and **b** per cubic centimetre TR. Values given as mean ± SD. *Bars topped by the same letter* do not differ significantly at *P* ≤ 0.05 (*n* = 4)

In the treatment with AMF (AM_v_), AMF hyphae were present along most of the length (55–80 %) of TR originating from all four plant species tested (Fig. 3a). Between 15 and 30 % of the TR length from nasturtium and tomato (both genotypes) also harboured storage organs intraradically (Fig. 3a). TR originating from pak choi contained a significantly lower percentage of intraradical storage organs of the TR length (up to 12 %) than the other TR sources. This was also reflected in a significantly higher hyphae-to-storage organ TR length ratio for pak choi TR compared to other TRs (Fig. 3b). Similarly, the number of storage organs per unit TR dry weight was significantly smaller in the pak choi TR compared with other TRs (Fig. 4a). Translated to unit of TR volume, high numbers (several thousands) of intraradical storage organs were present. In this respect, the storage organ quantity found in nasturtium TR was about three times higher than in pak choi TR; the counts for both tomato TR species were intermediate (Fig. 4b).

### AMF structure abundance in the TR diameter size classes

**Fig. 5:**
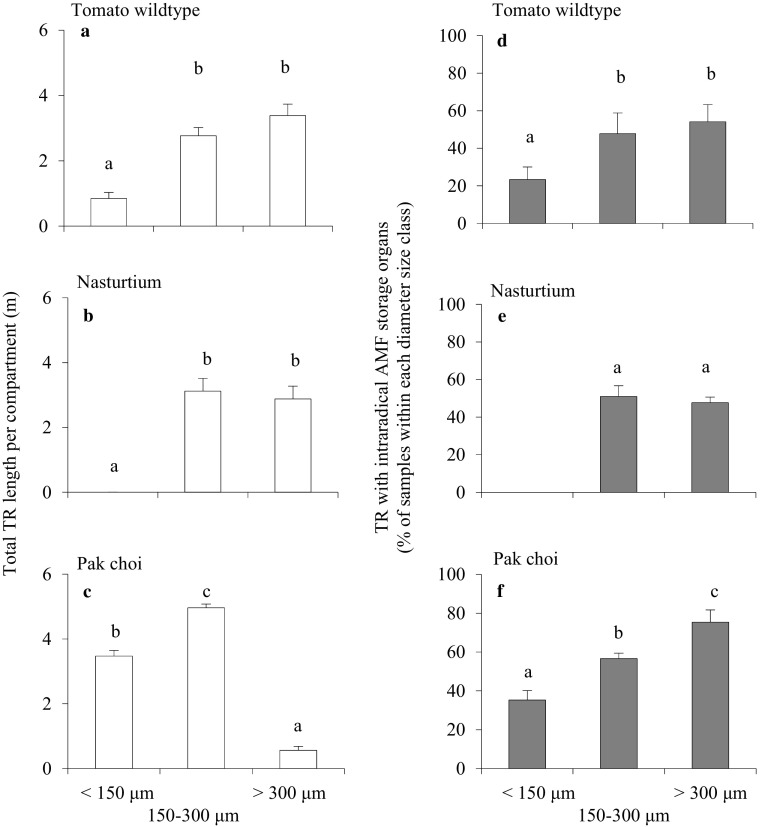
Total length of TR per compartment (in metre) of the different diameter size classes (**a**, **b**, **c**) and TR with intraradical AMF storage organs in percent of samples within each diameter size class (**d**, **e**, **f**). Values were given as mean ± SD. *Bars topped by the same letter* do not differ significantly at *P* ≤ 0.05 (*n* = 4)

The TR geometry (i.e. diameter size distribution) among the different TR origins (plant species) was heterogeneous (Fig. 5a, b, c); tomato TR predominantly consisted of diameters larger than 150 μm, and very few of the TR fell in the ‘thin’ category (Fig. 5a); in nasturtium TR samples, thin roots were completely absent (Fig. 5b). In contrast, the TR originating from pak choi included three times higher TR length of the <150 μm diameter size class than of the coarse >300 μm size class (Fig. 5c). Figure 5 shows that, in both pak choi and tomato, the percentage of TR length harbouring intraradical storage organs was significantly higher in the >300 μm class than in the <150 μm class. Trap root diameter, number of AMF storage organs per unit trap root length and the TR with intraradical AMF storage organs in percent of samples within each diameter size class were not significantly different between the two tomato genotypes. Therefore, only results from the wildtype tomato plants are shown in Fig. 5.

### Maize plant root biomass, AMF colonisation and spore number in the substrate

The maize root dry weight was 4.2 ± 0.8 g and the percentage of the total root length colonised by AMF was 83 ± 15 %, with 24 ± 6 % containing arbuscules, and 20 ± 5 % containing intraradical vesicles. The spore number within the growth substrate was 51 ± 11 spores per cubic centimetre.

## Discussion

In the 14-day period of trap root (TR) exposure to AMF-inoculated plants, AMF mycelium and storage organs developed on the outer surface of and within the TR. The fact that no AMF structures developed in TR exposed to the ‘maize shoot removal’ treatment showed that the AMF structures present in the TR after the 14-day period exposure to mycorrhizal plants were newly produced by *F. mosseae*.

According to the length of incubation period of TR, the AMF structures cannot have been older than 14 days, a period likely insufficient, for example, for spores to reach full maturity. This possibly caused the ubiquitous smaller diameter of storage organs inside of TRs compared with common spore sizes of *F. mosseae*. For example, Giovannetti et al. (2003) measured average spore diameters of 150 and 250 μm for different *F. mosseae* isolates. In terms of shape and size, AMF spores are sometimes not clearly distinct from vesicles, and a morphological differentiation between spores and vesicles was not attempted in the present experiment. Therefore, the storage organs quantified within TRs were assumed to include juvenile spores and/or vesicles. Concerning the re-colonisation potential of AMF-colonised TR, it will be necessary to study in a range of AMF species whether vesicles or spores are formed in the TR. For example, species of *Gigaspora* do not use intra- or extraradical vesicles as infective units (Biermann and Lindermann 1983; Klironomos and Hart 2002). In case mainly vesicles and juvenile spores are present in TR, the inoculum will have less infectivity than inoculum consisting of mature spores. Therefore, to improve their infectivity, the TRs should be exposed to AMF-inoculated plants for longer time periods. Marleau et al. (2011) demonstrated that the spore size continuously increases during the maturing process of about 30 days and about that time period is needed for spores to germinate and to serve as propagules. The TR technique was studied here on the basis of *F. mosseae*, known to form spores and vesicles. In order to fully understand AM fungal colonisation strategies in this regard, future studies should include other members of the family *Glomeraceae* as well as the large spectrum of orders within the *Glomeromycetes*.

Although hyphal growth was clearly present in the nylon mesh trap compartments, storage organs were always absent therein. A high number was harboured in most of the tested TR, e.g. 1 mg of TR sourced from tomato plants contained a similar quantity of intraradical storage organs as one cubic centimetre (equal to 1300 mg) of surrounding substrate. ERM proliferation and storage organ formation of AMF is known to be enhanced where the growth medium provides plenty of organic matter (Quilliam et al. 2010). With the beginning of its mineralisation, the release of nutrients from soil organic matter is rapid and initialised within a few days (Nett et al. 2015), allowing nutrients to be readily taken up by the ERM. This has been shown for nitrogen (Hodge and Fitter 2010) and phosphorus (Duan et al. 2011). The most important nutrient in this context is nitrogen. In in vitro cultures, Bago et al. (2004) have shown that ERM growth and spore formation are promoted by the supply of nitrate. AMF also transfer considerable amounts of nitrogen from decaying roots to the host plant via the ERM (Müller et al. 2012). However, the contribution of organic matter to AMF storage organ formation remains controversial, possibly reflecting the variability of compositions of the organic matter used in the different experiments (Gryndler et al. 2009). The presence of both saprophytic fungi (Ames et al. 1989) and AMF spore-associated bacteria (Mayo et al. 1986) has a positive effect on AMF ERM growth. Although small quantities of cellulolytic and other hydrolytic enzymes have been associated with AMF-colonised roots (Garcia-Garrido et al. 2000), their origin has not been definitely attributed to the AMF (Morales Vela et al. 2007). Many *Basidiomycota* and *Ascomycota* which are common in the rhizosphere produce enzymes able to degrade lignin and/or cellulose (Osono and Takeda 2006), but still there is no evidence that AMF might forage for nutrients from organic matter in the way saprophytic fungi do.

The percentage of trap root length with intraradical storage organs was significantly lower in TR derived from pak choi compared with the TRs from the other sources especially from tomato, its *rmc* mutant and nasturtium. The roots of both pak choi and nasturtium contain significant quantities of glucosinolates (Verkerk et al. 2009), compounds supposed to act as repellents against insects and phytopathogens (Bones and Rossiter 1996). Glucosinolate-derived isothiocyanates have known fungitoxic activity (Schreiner and Koide 1993). They are synthesised when glucosinolates are exposed to myrosinases following damage to cells (Bones and Rossiter 1996). It is possible that these compounds were present in the pak choi and nasturtium TRs used here, since cell walls were surely damaged in the chopped TR tissue. Though high contents of benzylglucosinolate are present in roots of nasturtium, it represents an AMF host plant (Ludwig-Müller et al. 2002), since root cells are not injured during AMF colonisation. Nevertheless, the present study showed that excised roots from this species supported formation of AMF storage organs just as TR from the non-glucosinolate producing tomato. The initial glucosinolate content of crucifer tissue mixed with soil substrate was shown to be degraded to a large extent after 4–6 days, depending on soil conditions (Bending and Lincoln 1999). Although membrane bags prevented TR from direct contact with the growth substrate, it is possible that, also in the present study, glucosinolates may have been degraded within the first half of the incubation period. Thus, the poor performance of the pak choi TR may be unrelated to presence of glucosinolates. It may rather reflect the cellular geometry of pak choi roots, characterised by smaller average diameter and cell size (as observed by microscopy) compared with the other tested TR sources.

Since TR diameter is a measure of the space available for the deposition of AMF structures per unit root length, it was obvious to probe the relationship between number of storage organs and TR diameter. In TR samples where a range of root diameter size was represented, the proportion of the TR length containing storage organs was considerably higher in coarse than in thin TRs. Unlike the typical symbiotic colonisation pattern, where AMF develop structures solely within the root cortex (Sieverding 1991), here, the AMF storage organs were also present in the vascular cylinder. The latter structures provide an extensive air space within the TR tissue, which may be particularly attractive as a site for storage organ accumulation due to the air-filled voids. Accordingly, using decomposing plant leaves, another study provided evidence that AMF structures preferably colonise tissue with large lumen such as the vascular tissue (Aristizábal et al. 2004). In roots of plants that were grown under hypoxic conditions, air spaces in the cortex (aerenchyma) can reach at least 100 μm in diameter; those compartments also hold considerable volumes of air in the intercellular space of the cortex (Evans 2004). TR materials used in the present study have experienced a degree of cell collapse as they were excised and it is likely that they too have included air spaces within the cortex and also inside of the vascular cylinders. These spaces are possibly detectable by AMF during ERM growth and provide an opportunity for the AMF to form storage organs therein. In this regard, due to their geometry, TR from pak choi are likely less appropriate for AMF penetration and formation of intraradical storage organs compared with other tested TR sources.

The interior of dead plant roots represents a favourable environment for the formation of storage organs of *F. mosseae*, which may be preferred over air spaces in the growth substrate. Irrespective of originating from a host or non-host species, annual plant species with large diameter roots may be highly suitable as a medium for the embedding of AMF spores and vesicles. The TR technique described here provides a means to generate AM fungal structures free of mineral substrates, which may facilitate the preparation of AMF for convenient microscopic and physiological analyses.
